# Preoperative prediction of HCC microvascular invasion by multimodel peritumoral diffusion MRI

**DOI:** 10.1186/s41747-026-00817-9

**Published:** 2026-10-23

**Authors:** Yunfei Zhang, Jiejun Chen, Xianling Qian, Mengsu Zeng

**Affiliations:** 1 Department of Radiology, Zhongshan Hospital, Fudan University, Shanghai, China; 2 Shanghai Institute of Medical Imaging, Fudan University, Shanghai, China

**Keywords:** Carcinoma (hepatocellular), Diffusion magnetic resonance imaging, Magnetic resonance imaging, Neoplasm invasiveness, Tumor microenvironment

## Abstract

**Objective:**

Most studies emphasize intratumoral diffusion-weighted imaging (DWI) metrics, but few studies have examined whether peritumoral DWI metrics can provide added value. We aimed to evaluate three-dimensional, multiscale, multimodel DWI parameters within intratumoral and peritumoral volumes of interest for predicting microvascular invasion (MVI) in hepatocellular carcinoma (HCC).

**Materials and methods:**

Seventy patients with HCC, including 42 with MVI, were prospectively enrolled. We calculated parameters from monoexponential, intravoxel incoherent motion, diffusion kurtosis imaging (DKI), stretched-exponential model (SEM), and continuous-time random-walk models within the tumor and in 3-mm (P3) and 5-mm (P5) peritumoral rims. Features showing differences on univariable analysis were further screened using logistic regression with variance inflation factor filtering to build combined models. Diagnostic performance was assessed by areas under the receiver operating characteristic curve (AUROC).

**Results:**

Four intratumoral diffusion-coefficient–related metrics showed significant differences between groups. In the peritumoral regions, 12 parameters differed between groups, including diffusion-coefficient–related indices and heterogeneity-related metrics. Among single parameters, P3_DKI_*K*_app_ achieved the best discrimination for MVI (AUROC = 0.713); P5_DKI_*K*_app_ and Tumor_SEM_DDC were selected as independent imaging predictors. After integrating alpha-fetoprotein (AFP) and tumor size, the combined model (Cli-IntraPeriDWI) yielded the best performance (AUROC = 0.844). SHAP analysis indicated that higher AFP, larger tumor size, higher P5_DKI_*K*_app_, and lower Tumor_SEM_DDC were associated with increased MVI risk.

**Conclusion:**

Peritumoral DWI offers multiple biomarkers and outperforms intratumoral metrics for MVI prediction. An interpretable model integrating peritumoral and intratumoral DWI with clinical factors enables accurate, noninvasive preoperative MVI risk stratification in HCC.

**Key Points:**

***Question*** Can multiscale, multimodel peritumoral DWI metrics improve preoperative prediction of MVI in HCC compared with intratumoral DWI metrics?

***Findings*** Compared with intratumoral DWI, peritumoral DWI offered more potential imaging biomarkers and superior diagnostic capability for the preoperative diagnosis of MVI in HCC.

***Relevance statement*** By integrating intratumoral and peritumoral metrics with clinical risk factors, we developed a model achieving optimal performance and transparent decision interpretability for MVI assessment, supporting preoperative prognostic evaluation and individualized treatment planning in HCC.

**Graphical Abstract:**

**Figure:**
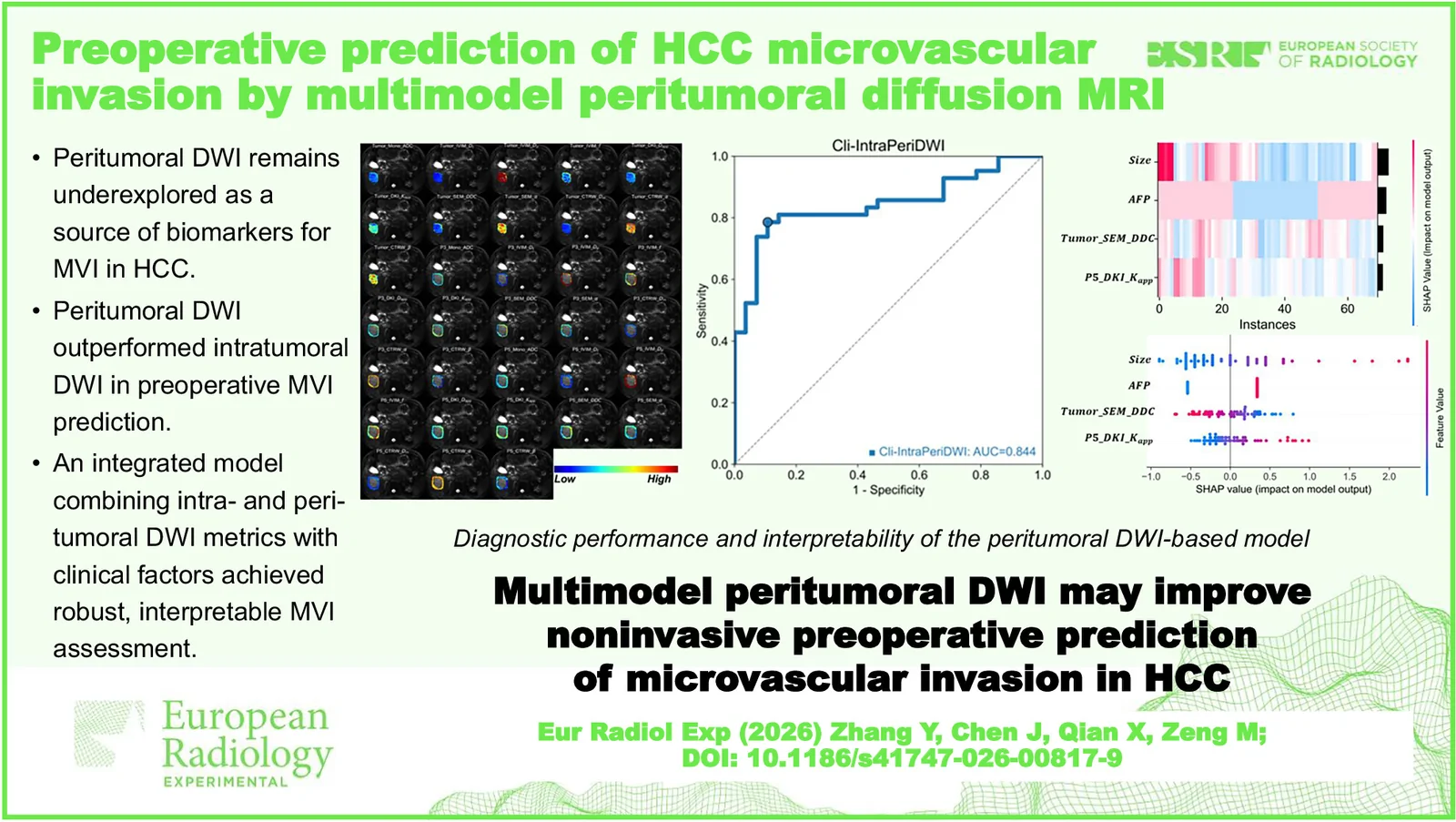

## Background

Diffusion-weighted imaging (DWI) provides a unique window into tissue microstructure by characterizing the diffusion behavior of water molecules [1–3]. Among its diverse applications, oncologic imaging represents one of the most prominent. Over the past decades, DWI has shown great promise in tumor detection, grading, therapeutic monitoring, and prognostic assessment [4]. Beyond the conventional mono-exponential DWI model, which uses the apparent diffusion coefficient (ADC) as a surrogate of cellularity, a spectrum of advanced diffusion models—such as intravoxel incoherent motion (IVIM) [5], diffusion kurtosis imaging (DKI) [6], stretched-exponential model (SEM) [7], fractional-order calculus [8], restriction spectrum imaging [9], mean apparent propagator MRI [10], and continuous-time random walk (CTRW) [11]—has been developed to capture more biologically specific diffusion characteristics. These models expand the diagnostic landscape from simple cellularity estimation to multidimensional characterization of vascularity, microstructural complexity, and restricted diffusion, offering more comprehensive insights into tumor biology.

Traditionally, quantitative analysis of DWI-derived parameters within two-dimensional regions of interest (ROIs) or three-dimensional volumes of interest (VOIs) has been the primary approach for identifying potential imaging biomarkers. Such analyses have revealed key intratumoral features, including increased cellularity, vascularity, and heterogeneity [4]. However, tumors represent highly complex “ecosystems” whose invasion and progression involve not only intrinsic changes but also dynamic alterations in the peritumoral microenvironment—encompassing hemodynamics, stromal remodeling, inflammatory response, and infiltration pattern [12, 13]. These peritumoral changes often precede or exceed volumetric progression, carrying independent diagnostic and prognostic significance. Indeed, peritumoral microenvironment alterations manifest as recognizable imaging features which have been incorporated into major diagnostic frameworks including Liver Imaging Reporting and Data System (LI-RADS) [14]. Yet, few studies have systematically explored whether peritumoral DWI-based quantitative metrics could serve as imaging biomarkers. Most existing work relies on manually placed two-dimensional ROIs or three-dimensional VOIs for ADC measurement, which are prone to sampling bias and operator dependence, and rarely consider (1) the multi-scale nature of peritumoral effects, (2) integrated three-dimensional analyses of the peritumoral region, or (3) cross-model evaluations among advanced DWI techniques such as IVIM, DKI, or time-dependent DWI.

Hepatocellular carcinoma (HCC) remains among the leading causes of cancer-related mortality, and microvascular invasion (MVI) is a critical prognostic factor associated with poor outcomes [15]. As a peritumoral pathological hallmark, MVI provides an ideal biological anchor for investigating the diagnostic value of peritumoral DWI-derived parameters. Furthermore, some previous investigations have highlighted the role of peritumoral MRI features in identifying MVI [16–18]. Therefore, this study aims to evaluate multi-scale peritumoral quantitative metrics derived from mono-exponential DWI, IVIM, DKI, SEM, and CTRW models to determine their potential as imaging biomarkers for the preoperative prediction of MVI in HCC.

## Materials and methods

### Power analysis

Prior to initiating this prospective study, a power analysis based on receiver operating characteristic (ROC) curve evaluation was performed to estimate the required sample size [19]. Assuming a predefined area under the receiver operating characteristic curve (AUROC) of 0.70, a significance level of 0.05, and a statistical power of 0.80, the minimum required sample size was calculated to be 62. This study was approved by the Ethics Committee of Zhongshan Hospital, Fudan University, Shanghai, China [approval number: B2020-372R], and written informed consent was obtained from all participants before enrollment. All procedures were conducted in accordance with the Declaration of Helsinki.

### Inclusion and exclusion criteria

We included patients who were clinically or radiologically suspected, or preliminarily diagnosed, as having HCC based on clinical risk factors, alpha-fetoprotein (AFP) and other laboratory findings, and typical imaging features on dynamic contrast-enhanced CT/MRI, with reference to LI-RADS v2018; all included patients were ultimately confirmed as having HCC by postoperative histopathology.

We excluded: (1) pathologically confirmed liver malignancies other than HCC (*e.g*., intrahepatic cholangiocarcinoma); (2) any prior liver-directed antitumor therapeutic intervention before MRI examination; (3) an interval longer than one month between MRI examination and surgical resection; (4) poor image quality on MRI precluding further analysis; and (5) unavailable pathological report for MVI assessment.

### MRI protocol

All MRI examinations were performed using a 3-T commercial scanner (Shanghai United Imaging Healthcare Co., Ltd., Shanghai, China). A customized multi-*b*-value DWI sequence based on spin-echo echo-planar imaging with spectral fat suppression was applied under free-breathing acquisition. Standard vendor reconstruction, including system-related geometric correction when available, was applied, and multiple signal averages were used across *b*-values to reduce respiratory motion-related signal fluctuation; no additional offline motion correction was performed. The main imaging parameters were as follows: repetition time/echo time 3,800/75 ms; flip angle 90°; field of view 300 × 380 mm²; matrix 202 × 256; and slice thickness 6 mm. The diffusion gradients were applied along three orthogonal directions, with ten *b*-values of 0₁, 20₁, 40₁, 50₁, 100₂, 200₂, 500₃, 800₃, 1,500₄, and 2,000_5_ s/mm² (subscripts denote the number of signal averages). The total acquisition time for the DWI sequence was approximately 3.5 min. Routine liver MRI sequences were also acquired, with detailed parameters provided in the Supplementary Materials.

### MVI reference standard

MVI status was determined by histopathological assessment of surgically resected specimens and was classified as either MVI-positive or MVI-negative. MVI was defined as the presence of tumor cell nests or emboli within an endothelium-lined vascular lumen under microscopy, typically located in the tumor capsule or adjacent liver parenchyma; pathologists were blinded to the DWI-derived parameters and model predictions.

### Data analysis

Two observers participated in VOI delineation. One observer was an abdominal radiologist with 5 years of experience, and the other was an assistant researcher with 8 years of experience in HCC–related MRI research, prior radiologic diagnostic training, and extensive experience in HCC ROI/VOI delineation. Tumor VOIs were manually delineated using ITK-SNAP software (version 3.8.0, https://www.itksnap.org/pmwiki/pmwiki.php) on DWI images with a *b*-value of 0 s/mm² to encompass the entire HCC lesion. During VOI delineation, both observers were aware of the lesion location and the diagnosis of HCC to ensure accurate tumor identification and segmentation but were blinded to the pathological MVI status and other clinical outcome information. The VOI delineation was performed using a consensus-based workflow. Specifically, the two observers reviewed the MR images together and delineated the tumor boundary slice by slice through discussion. Any disagreement regarding lesion boundaries was resolved by consensus under the supervision of a senior radiologist with more than 30 years of experience in abdominal imaging. Therefore, the final VOI used for analysis represented a consensus VOI rather than one derived from two independent segmentations.

Furthermore, peritumoral VOIs corresponding to 3 mm and 5 mm peritumoral regions (denoted as P3 and P5) surrounding the tumor were automatically generated using an in-house Python script based on three-dimensional morphological dilation, where the dilation size was defined in millimeters. The 3-mm and 5-mm rims were selected to balance sensitivity to MVI-related peritumoral changes with technical robustness, because overly narrow rims may be vulnerable to boundary uncertainty and partial-volume effects, whereas overly wide rims may dilute relevant signals by including distant normal liver parenchyma or adjacent large vessels/bile ducts.

In this study, voxel-wise diffusion parameters within each VOI were calculated using five diffusion models: mono, IVIM, DKI, SEM, and CTRW. Voxel-wise diffusion parameters were estimated using the model equations provided in the Supplementary Materials. Parameter fitting was implemented in Python (https://www.python.org/) using NumPy, SciPy, and SimpleITK, and model fitting was performed using nonlinear least-squares estimation. The mathematical formulas, *b*-values used for model fitting, parameter definitions, and principal biophysical interpretations of each diffusion model are provided in the Supplementary materials.

In brief, the monoexponential model generated the ADC. The IVIM model generated the true diffusion coefficient (${D}_{t}$), pseudo-diffusion coefficient (${D}_{{{\rm{p}}}}$), and perfusion fraction (f). The DKI model generated the apparent diffusion coefficient (${D}_{{\mathrm{app}}}$) and apparent kurtosis coefficient (${{{\rm{K}}}}_{{{\rm{app}}}}$). The SEM generated the distributed diffusion coefficient (${{\rm{DDC}}}$) and diffusion heterogeneity index (${{\rm{\alpha }}}$). The continuous-time random-walk model generated the generalized diffusion coefficient (${D}_{{{\rm{m}}}}$) and the temporal and spatial fractional exponents, ${{\rm{\alpha }}}$ and ${{\rm{\beta }}}$, respectively.

In summary, for each diffusion model–derived parameter, the mean value was calculated within three distinct VOIs: the tumor, P3, and P5. Taking the ADC from the Mono model as an example, three parameters were obtained: Tumor_Mono_ADC, P3_Mono_ADC, and P5_Mono_ADC. Other parameters were computed and labeled following the same convention, using the format spatial region_model name_parameter name.

### Statistical analysis

We first performed Shapiro–Wilk tests to assess the normality of all quantitative parameters. According to the distribution characteristics, univariable analysis was conducted using either the Student *t*-test or the Mann–Whitney *U*-test to determine whether significant differences existed between the MVI-positive and MVI-negative groups. For DWI-derived parameters that exhibited significant intergroup differences, a stepwise binary logistic regression was subsequently applied to identify independent imaging predictors. Variables with multicollinearity (variance inflation factor [VIF] > 5) were excluded to ensure model robustness and retain only potential independent imaging predictors. In parallel, univariate and multivariate logistic regression analyses were performed to determine independent clinical risk factors associated with MVI status. Based on the selected DWI parameters and clinical variables, a clinical-only model (Cli) including tumor size and AFP, an imaging-only model (IntraPeriDWI) including Tumor_SEM_DDC and P5_DKI_*K*_app_, and a combined clinical, intratumoral, and peritumoral diffusion-weighted imaging model (Cli-IntraPeriDWI) integrating tumor size, AFP, Tumor_SEM_DDC, and P5_DKI_*K*_app_ were constructed. The diagnostic performance of both individual predictors and the combined model was evaluated using ROC curve analysis, including the area under the curve (AUROC), sensitivity, specificity, and their 95% confidence intervals (CIs). To further interpret the model’s decision-making process, SHapley Additive exPlanations (SHAP) analysis was employed to quantify the contribution of each feature to the Cli-IntraPeriDWI model. Statistical power analyses were performed using PASS software (version 2021; NCSS, Kaysville, Utah, USA), while all other statistical procedures were implemented via customized Python scripts. A two-tailed *p*-value < 0.05 was considered to indicate statistical significance.

## Results

### Study cohort

**Fig. 1:**
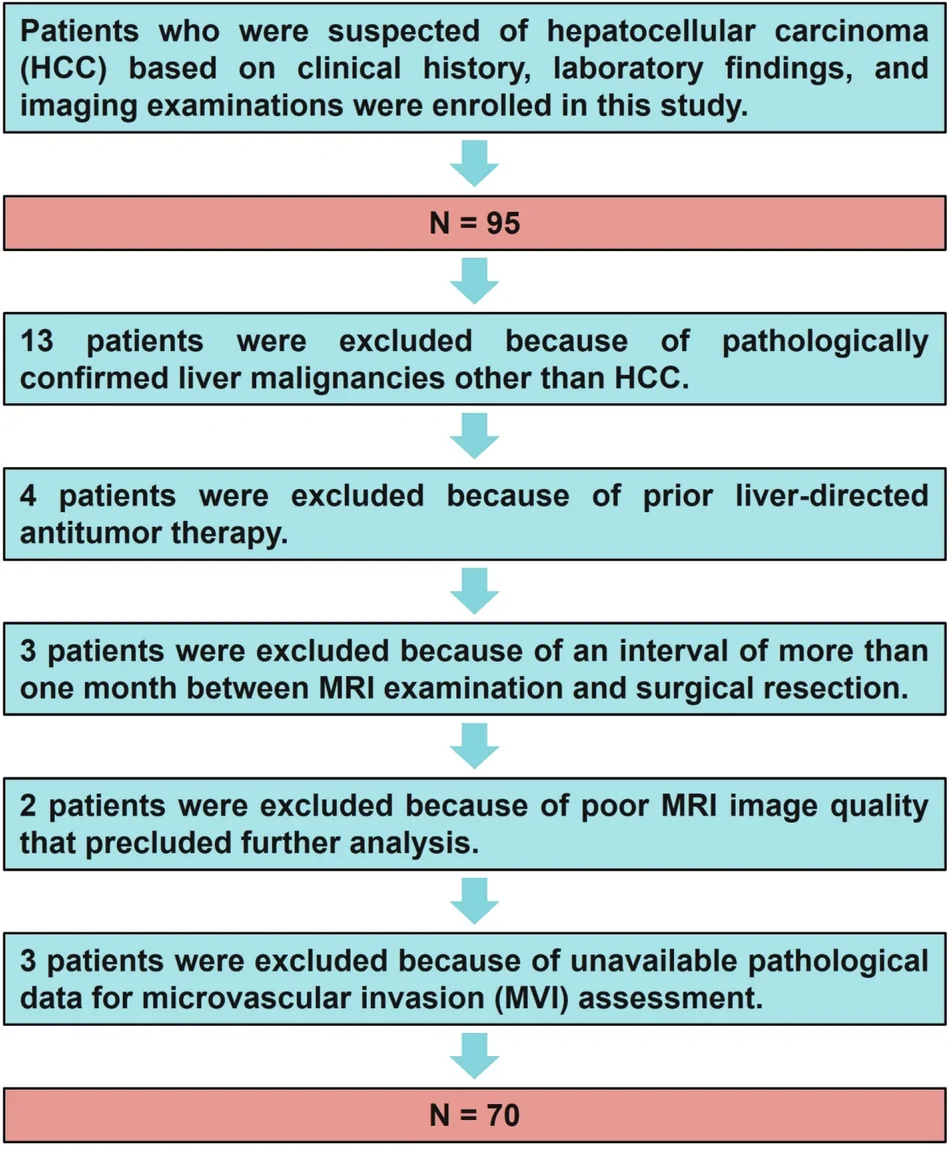
Study flow diagram depicting patient screening, exclusions, and final cohort included in the analysis

As shown in Fig. 1, a total of 95 patients with suspected HCC were initially enrolled in this prospective study. After sequential exclusion, 25 patients were excluded for the following reasons: pathologically confirmed non-HCC (*n* = 13), prior liver-directed antitumor therapy (*n* = 4), excessive interval between MRI examination and surgical resection (*n* = 3), poor image quality due to artifacts, low signal-to-noise ratio, or geometric distortion (*n* = 2), and lack of available histopathological diagnosis of MVI (*n* = 3). Ultimately, 70 patients (61 males, 42 MVI-positive; age 55.6 ± 10.6 years, mean ± standard deviation) were included in the final analysis. Detailed clinical and baseline characteristics are summarized in Table 1.

**Table 1:** Baseline clinical and pathological characteristics of patients with MVI-positive HCC and MVI-negative HCC (*n*** = **70)

Characteristic	Category	MVI-positive (*n* = 42)	MVI-negative (*n* = 28)
Age	Mean ± SD	54.10 ± 10.07	57.75 ± 11.07
	Range	(33–73)	(36–83)
Gender	Female	19.05% (8/42)	3.57% (1/28)
	Male	80.95% (34/42)	96.43% (27/28)
TBil	Normal (≤ 20.4 μmol/L)	85.71% (36/42)	78.57% (22/28)
	Elevated (> 20.4 μmol/L)	14.29% (6/42)	21.43% (6/28)
DBil	Normal (≤ 6.8 μmol/L)	90.48% (38/42)	78.57% (22/28)
	Elevated (> 6.8 μmol/L)	9.52% (4/42)	21.43% (6/28)
ALT	Normal (≤ 35 U/L)	76.19% (32/42)	75.00% (21/28)
	Elevated (> 35 U/L)	23.81% (10/42)	25.00% (7/28)
AST	Normal (≤ 40 U/L)	80.95% (34/42)	78.57% (22/28)
	Elevated (> 40 U/L)	19.05% (8/42)	21.43% (6/28)
GGT	Normal (≤ 60 U/L)	57.14% (24/42)	71.43% (20/28)
	Elevated (> 60 U/L)	42.86% (18/42)	28.57% (8/28)
AFP	Negative (≤ 20 ng/mL)	23.81% (10/42)	60.71% (17/28)
	Positive (> 20 ng/mL)	76.19% (32/42)	39.29% (11/28)
CEA	Negative (< 5 ng/mL)	92.86% (39/42)	92.86% (26/28)
	Positive (≥ 5 ng/mL)	7.14% (3/42)	7.14% (2/28)
CA19-9	Negative (< 37 U/mL)	76.19% (32/42)	85.71% (24/28)
	Positive (≥ 37 U/mL)	23.81% (10/42)	14.29% (4/28)
Etiology	Hepatitis B virus (HBV)	97.62% (41/42)	100.00% (28/28)
	Hepatitis C virus (HCV)	0.00% (0/42)	7.14% (2/28)
Cirrhosis	Absent	30.95% (13/42)	46.43% (13/28)
	Present	69.05% (29/42)	53.57% (15/28)
Size (cm)	Mean ± SD	5.82 ± 3.64	3.79 ± 1.75
	Range	(1.0–15.0)	(1.0–7.0)
Grade	Low grade	57.14% (24/42)	85.71% (24/28)
	High grade	42.86% (18/42)	14.29% (4/28)

HCC grade was assessed according to the Edmondson–Steiner grading system

Continuous variables are presented as mean ± standard deviation (range), and categorical variables are presented as frequency and percentage (*n*, %). Laboratory variables were categorized according to the following cutoff values: TBil, normal ≤ 20.4 μmol/L and elevated > 20.4 μmol/L; DBil, normal ≤ 6.8 μmol/L and elevated > 6.8 μmol/L; ALT, normal ≤ 35 U/L and elevated > 35 U/L; AST, normal ≤ 40 U/L and elevated > 40 U/L; GGT, normal ≤ 60 U/L and elevated > 60 U/L; AFP, negative ≤ 20 ng/mL and positive > 20 ng/mL; CEA, negative < 5 ng/mL and positive ≥ 5 ng/mL; CA19-9, negative < 37 U/mL and positive ≥ 37 U/mL

*AFP* Alpha-fetoprotein, *HBV* Hepatitis B virus, *HCC* Hepatocellular carcinoma, *HCV* Hepatitis C virus, *ALT* Alanine aminotransferase, *AST* Aspartate aminotransferase, *TBil* Total bilirubin, *DBil* Direct bilirubin, *GGT* γ-glutamyl transferase, *MVI* Microvascular invasion

### Intergroup comparison

**Fig. 2:**
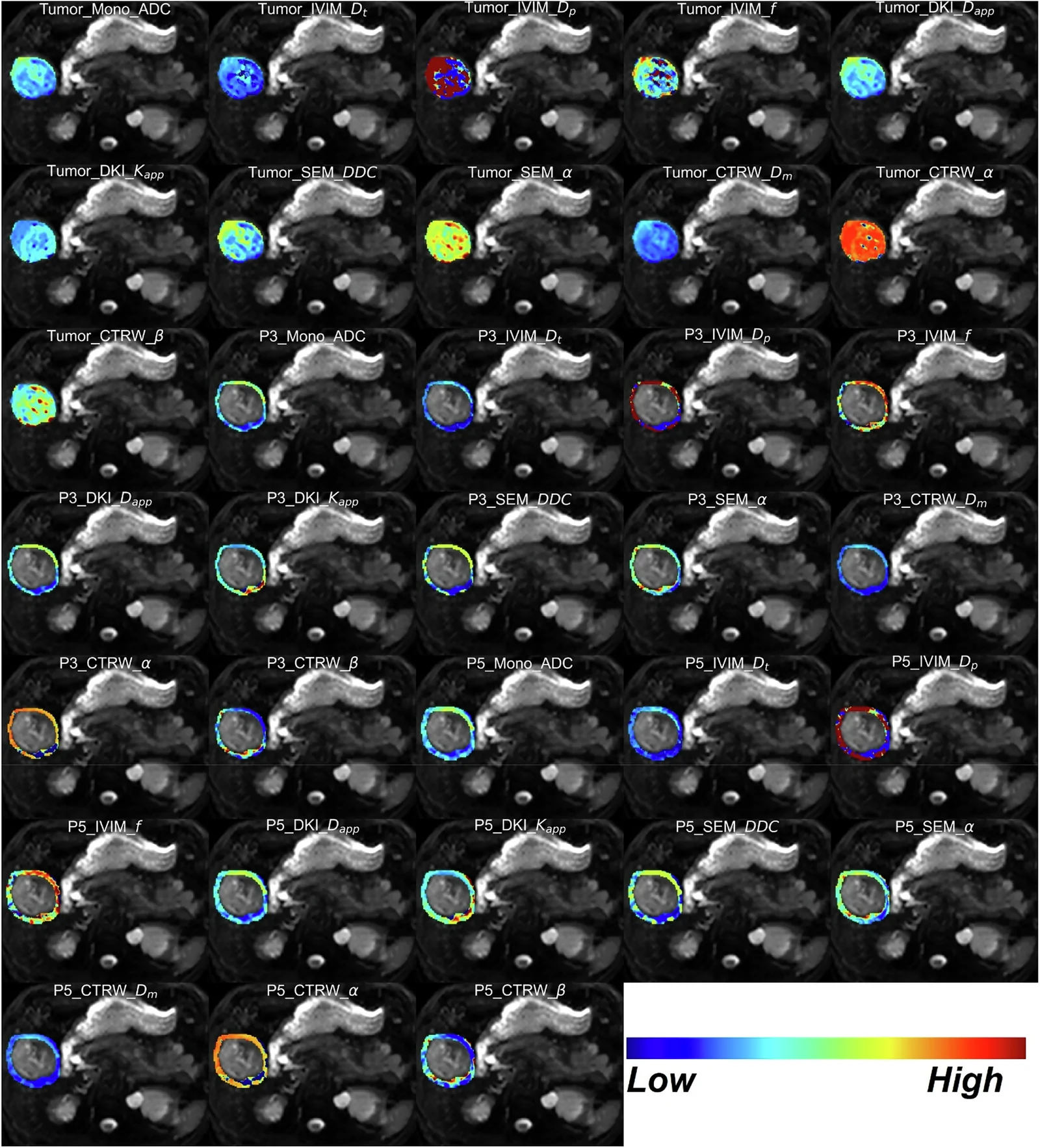
Representative multi-scale and multi-model DWI parametric maps from a male patient (63 years) with MVI-negative HCC. For each diffusion parameter, pseudo-color parametric maps are overlaid on the corresponding DWI image of *b* = 0 s/mm². Panel labels indicate the diffusion model, quantitative parameter, and spatial scale. For example, Tumor_Mono_ADC (row 1, column 1) denotes an ADC map derived from the mono-exponential model within the intratumoral region, while P3_DKI_*K*ₐₚₚ (row 4, column 2) represents a *K*ₐₚₚ map derived from the DKI model within the 3-mm peritumoral rim. All maps are overlaid on the same *b* = 0 image for reference. The color bar represents low-to-high parameter values from blue to red. Mono, Mono-exponential model; ADC, Apparent diffusion coefficient (Mono); IVIM, Intravoxel incoherent motion; *D*ₜ, True diffusion coefficient; *D*ₚ, Pseudodiffusion coefficient; *f*, Perfusion fraction; DKI, Diffusion kurtosis imaging; *D*ₐₚₚ, Apparent diffusion coefficient (DKI); *K*ₐₚₚ, Apparent kurtosis; SEM, Stretched-exponential model; DDC, Distributed diffusion coefficient; α (SEM), Diffusion heterogeneity index; CTRW, Continuous-time random walk model; *D*ₘ, Generalized diffusion coefficient; α (CTRW) and β (CTRW), Temporal and spatial fractional exponents; P3/P5, 3-mm/5-mm peritumoral rim

**Fig. 3:**
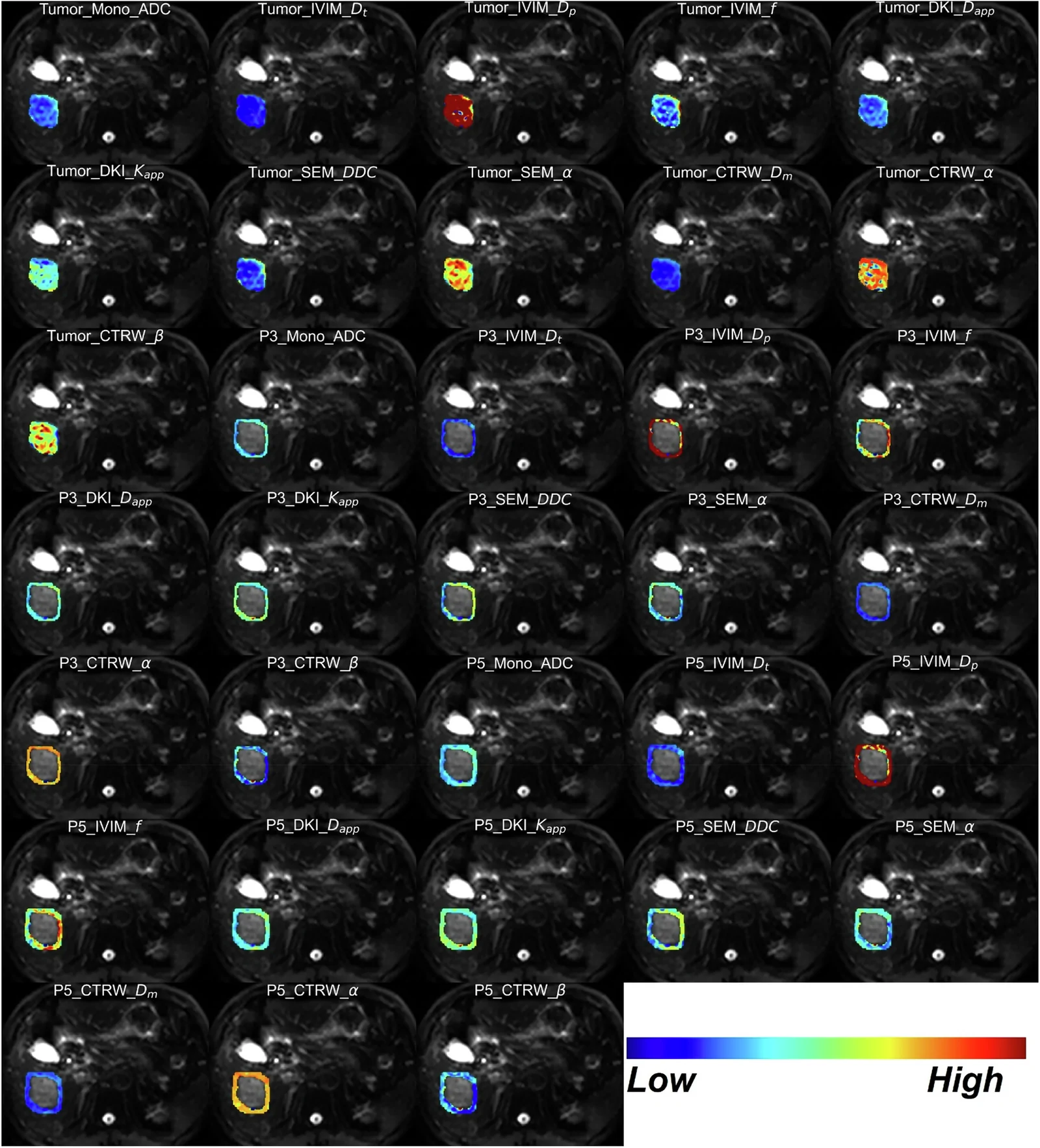
Another representative set of multi-scale, multi-model DWI parametric maps from a patient (male, 56 years) with MVI-positive HCC, displayed as in Fig. 2. Panel labels and abbreviation definitions are identical to those in Fig. 2

Figures 2 and 3 present two representative cases: one MVI-negative HCC in a 63-year-old male and one MVI-positive HCC in a 56-year-old male. Parametric pseudo-color maps of various DWI-derived metrics are displayed for both cases at different spatial scales, superimposed on the corresponding DWI images of *b* = 0 s/mm². As shown in Figures 2 and 3, the MVI-positive tumor exhibited lower intratumoral diffusion-related parameters (*e.g.*, Mono_ADC, DKI_*D*_app_, SEM_DDC), whereas the peritumoral region demonstrated higher kurtosis-related metrics (DKI_*K*_app_) and lower diffusion-related metrics (*e.g*., Mono_ADC, DKI_*D*_app_, SEM_DDC).

**Fig. 4:**
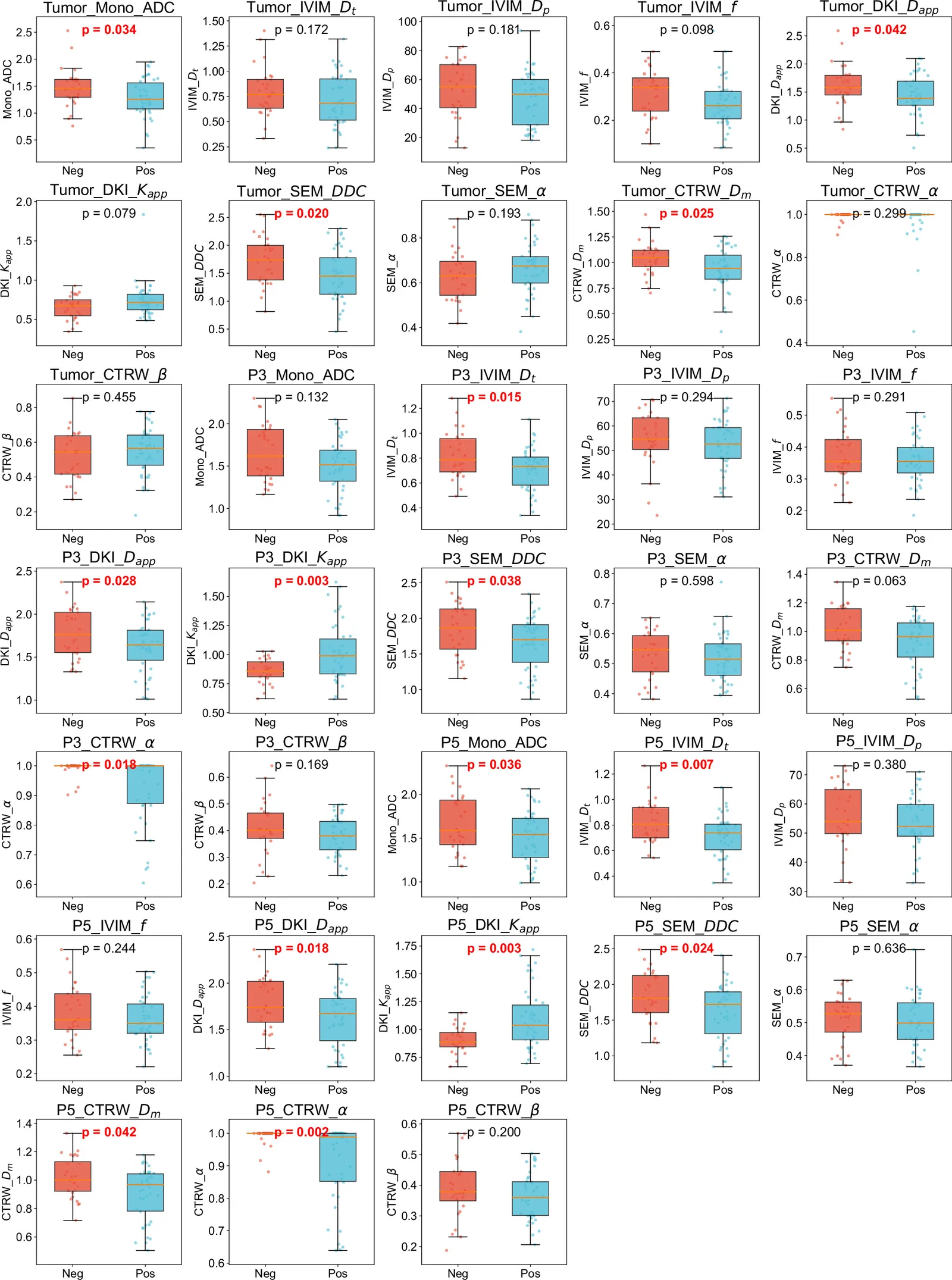
Boxplots show the differences in quantitative parameters between MVI-positive and MVI-negative groups. The *p*-values of group comparisons are displayed above each subplot. The *p*-values were obtained using either the Student's *t*-test or the Mann–Whitney *U*-test, with results of *p* < 0.05 highlighted in red. Parameter names follow the convention “SpatialScale_Model_Parameter.” For example, Tumor_Mono_ADC represents the mean ADC derived from the Mono model within the tumor region (Tumor). Peritumoral regions of 3 mm and 5 mm are denoted as P3 and P5, respectively; other parameters follow the same naming rule. Units: ADC, *D*ₜ, *D*ₚ, *D*ₐₚₚ, *D*ₘ, DDC: ×10⁻³ mm²/s; *f*: dimensionless (fraction); *K*ₐₚₚ: dimensionless (kurtosis); α (SEM), α (CTRW), and β (CTRW): dimensionless

Quantitative results are shown in Fig. 4 and Table 2. Compared with the MVI-negative group, the MVI-positive group exhibited significantly reduced intratumoral diffusion coefficients, including Tumor_Mono_ADC, Tumor_DKI_*D*_app_, Tumor_SEM_DDC, and Tumor_CTRW_*D*_m_ (*p* = 0.020–0.042). In the peritumoral regions, significant decreases were also observed in diffusion-related parameters (P3_IVIM_D_t,_ P3_DKI_*D*_app_, P3_SEM_DDC, P5_Mono_ADC, P5_IVIM_D_t_, P5_DKI_*D*_app,_ P5_SEM_DDC, P5_CTRW_*D*_m_), accompanied by marked alterations in heterogeneity-related metrics, such as elevated P3_DKI_*K*_app_, P5_DKI_*K*_app_, and decreased P3_CTRW_α, and P5_CTRW_α (*p* = 0.002–0.042). Additional parameters and intergroup comparisons are summarized in Table 2.

**Table 2:** Comparison of quantitative diffusion parameters between MVI-negative and MVI-positive groups

Variables	MVI-negative (mean ± SD)	MVI-positive (mean ± SD)	*p*-value
Tumor_Mono_ADC	1.470 ± 0.366	1.280 ± 0.355	**0.034**
Tumor_IVIM_*D*_t_	0.798 ± 0.243	0.714 ± 0.253	0.172
Tumor_IVIM_*D*_p_	52.975 ± 19.362	46.838 ± 18.114	0.181
Tumor_IVIM_*f*	0.313 ± 0.102	0.271 ± 0.101	0.098
Tumor_DKI_*D*_app_	1.619 ± 0.378	1.432 ± 0.366	**0.042**
Tumor_DKI_*K*_app_	0.652 ± 0.150	0.741 ± 0.214	0.079
Tumor_SEM_DDC	1.726 ± 0.432	1.466 ± 0.458	**0.020**
Tumor_SEM_α	0.632 ± 0.110	0.667 ± 0.112	0.193
Tumor_CTRW_*D*_m_	1.042 ± 0.165	0.937 ± 0.200	**0.025**
Tumor_CTRW_α	0.992 ± 0.022	0.969 ± 0.094	0.299
Tumor_CTRW_β	0.522 ± 0.140	0.547 ± 0.131	0.455
P3_Mono_ADC	1.663 ± 0.340	1.505 ± 0.300	0.132
P3_IVIM_*D*_t_	0.824 ± 0.186	0.716 ± 0.172	**0.015**
P3_IVIM_*D*_p_	54.555 ± 11.449	52.545 ± 10.002	0.294
P3_IVIM_*f*	0.378 ± 0.081	0.359 ± 0.070	0.291
P3_DKI_*D*_app_	1.782 ± 0.295	1.619 ± 0.298	**0.028**
P3_DKI_*K*_app_	0.857 ± 0.105	1.018 ± 0.248	**0.003**
P3_SEM_DDC	1.846 ± 0.368	1.655 ± 0.370	**0.038**
P3_SEM_α	0.530 ± 0.079	0.520 ± 0.080	0.598
P3_CTRW_*D*_m_	1.022 ± 0.148	0.918 ± 0.182	0.063
P3_CTRW_α	0.990 ± 0.027	0.925 ± 0.115	**0.018**
P3_CTRW_β	0.407 ± 0.103	0.378 ± 0.071	0.169
P5_Mono_ADC	1.676 ± 0.332	1.513 ± 0.301	**0.036**
P5_IVIM_*D*_t_	0.828 ± 0.165	0.716 ± 0.164	**0.007**
P5_IVIM_*D*_p_	55.690 ± 10.808	53.525 ± 9.512	0.380
P5_IVIM_*f*	0.384 ± 0.082	0.362 ± 0.070	0.244
P5_DKI_*D*_app_	1.797 ± 0.280	1.625 ± 0.298	**0.018**
P5_DKI_*K*_app_	0.906 ± 0.110	1.090 ± 0.272	**0.003**
P5_SEM_DDC	1.837 ± 0.357	1.625 ± 0.386	**0.024**
P5_SEM_α	0.512 ± 0.075	0.503 ± 0.078	0.636
P5_CTRW_*D*_m_	1.014 ± 0.144	0.903 ± 0.183	**0.042**
P5_CTRW_α	0.990 ± 0.028	0.913 ± 0.121	**0.002**
P5_CTRW_β	0.390 ± 0.098	0.363 ± 0.077	0.200

Values are presented as mean ± standard deviation (mean ± SD): *p*-values were calculated using the independent-samples *t*-test or Mann–Whitney *U*-test, as appropriate, based on data distribution. Statistically significant differences (*p* < 0.05) are shown in bold. Units: *ADC*, *D*ₜ, *D*ₚ, *D*ₐₚₚ, *D*ₘ, *DDC*: ×10⁻³ mm²/s; *f*: dimensionless (fraction); *K*ₐₚₚ: dimensionless (kurtosis); α (SEM), α (CTRW), and β (CTRW): dimensionless

### Diagnostic performance

**Fig. 5:**
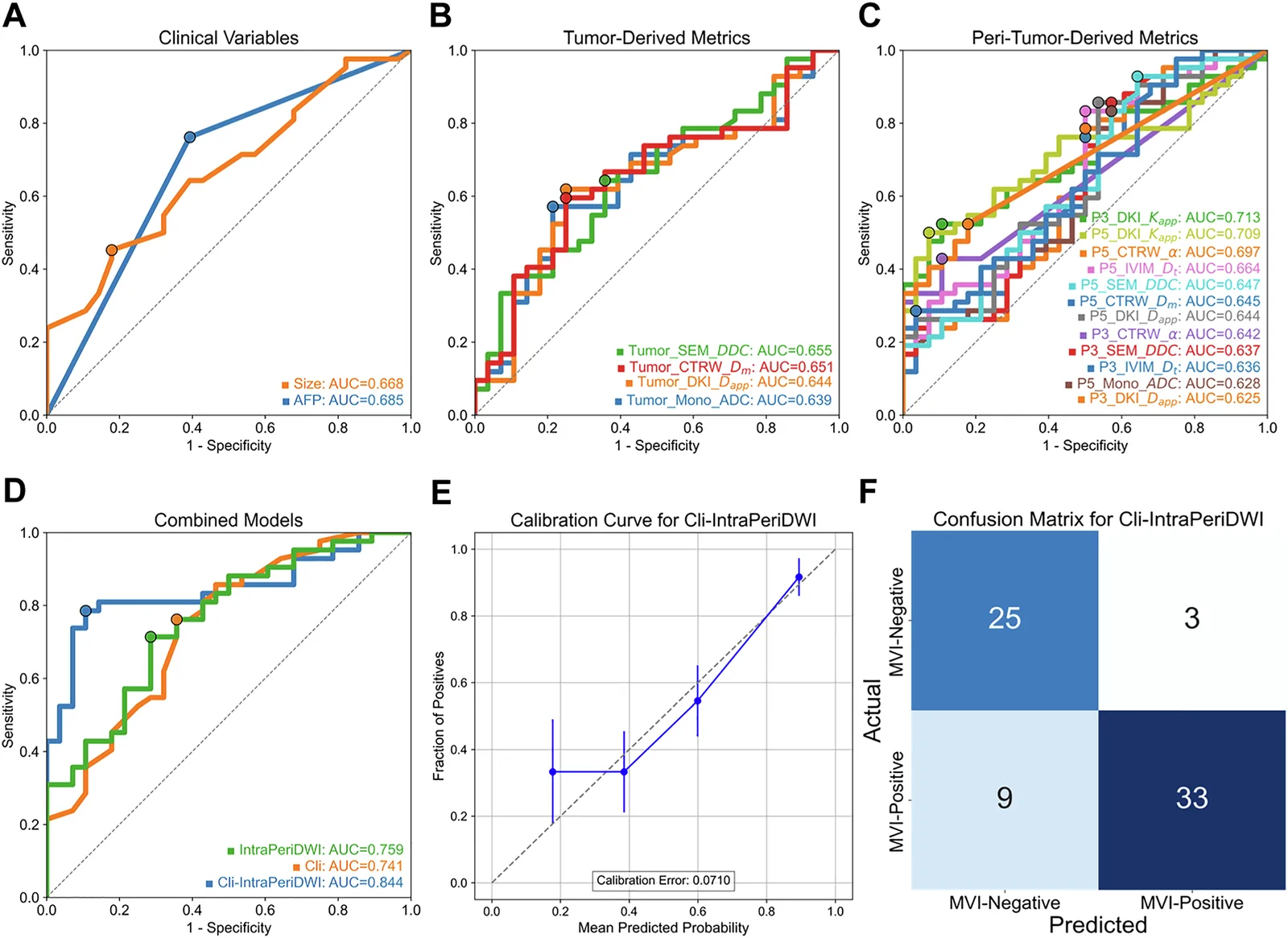
Diagnostic performance of clinical variables, DWI-derived metrics, and combined models for predicting MVI in HCC. **A** ROC curves of individual clinical variables, including tumor size and AFP. **B** ROC curves of representative intratumoral DWI-derived metrics, including Tumor_SEM_DDC, Tumor_CTRW_D_m_, Tumor_DKI_D_app_, and Tumor_Mono_ADC. **C** ROC curves of peritumoral DWI-derived parameters with significant intergroup differences. **D** ROC curves of the combined models. The Cli model included tumor size and AFP status; the IntraPeriDWI model included the selected independent imaging predictors, Tumor_SEM_DDC and P5_DKI_*K*_app_; and the Cli-IntraPeriDWI model integrated tumor size, AFP status, Tumor_SEM_DDC, and P5_DKI_*K*_app_. Colored circles indicate the optimal cutoff points determined using the Youden index. **E** Calibration curve of the Cli-IntraPeriDWI model. **F** Confusion matrix illustrating the classification performance of the Cli-IntraPeriDWI model

As shown in Fig. 5A and Table 3, among the clinical variables, AFP and tumor size showed moderate diagnostic performance for predicting MVI status, with AUROCs of 0.685 and 0.668, respectively. As shown in Fig. 5B, among intratumoral DWI parameters, Tumor_SEM_DDC yielded the highest diagnostic performance for predicting MVI (AUROC = 0.655), followed by Tumor_CTRW_D_m_, Tumor_DKI_D_app_, and Tumor_Mono_ADC.

**Table 3:** Diagnostic performance of clinical predictors, individual DWI-derived parameters, and combined models for predicting MVI

Metric	AUC (95% CI)	Sensitivity (95% CI)	Specificity (95% CI)
Size	0.668 (0.555–0.794)	0.452 (0.186–0.979)	0.821 (0.286–1.000)
AFP	0.685 (0.573–0.798)	0.762 (0.641–0.886)	0.607 (0.419–0.789)
Tumor_Mono_ADC	0.639 (0.519–0.780)	0.571 (0.326–0.811)	0.786 (0.518–0.957)
Tumor_DKI_*D*_app_	0.644 (0.518–0.778)	0.619 (0.375–0.810)	0.750 (0.555–0.955)
Tumor_SEM_DDC	0.655 (0.527–0.780)	0.643 (0.289–0.875)	0.643 (0.458–1.000)
Tumor_CTRW_*D*_m_	0.651 (0.527–0.784)	0.595 (0.349–0.837)	0.750 (0.500–0.967)
P3_IVIM_*D*_t_	0.636 (0.512–0.751)	0.762 (0.238–0.976)	0.500 (0.273–1.000)
P3_DKI_*D*_app_	0.625 (0.514–0.770)	0.786 (0.209–1.000)	0.500 (0.280–1.000)
P3_DKI_*K*_app_	0.713 (0.591–0.823)	0.524 (0.326–0.861)	0.893 (0.536–1.000)
P3_SEM_DDC	0.637 (0.518–0.766)	0.857 (0.239–0.979)	0.429 (0.292–1.000)
P3_CTRW_α	0.642 (0.539–0.746)	0.429 (0.225–0.579)	0.893 (0.857–1.000)
P5_Mono_ADC	0.628 (0.511–0.762)	0.833 (0.205–0.979)	0.429 (0.286–1.000)
P5_IVIM_*D*_t_	0.664 (0.526–0.789)	0.833 (0.270–0.977)	0.500 (0.318–1.000)
P5_DKI_*D*_app_	0.644 (0.516–0.773)	0.857 (0.233–0.976)	0.464 (0.346–1.000)
P5_DKI_*K*_app_	0.709 (0.576–0.820)	0.500 (0.343–0.837)	0.929 (0.630–1.000)
P5_SEM_DDC	0.647 (0.517–0.776)	0.929 (0.238–1.000)	0.357 (0.276–1.000)
P5_CTRW_*D*_m_	0.645 (0.520–0.766)	0.286 (0.213–1.000)	0.964 (0.258–1.000)
P5_CTRW_α	0.697 (0.595–0.794)	0.524 (0.250–0.667)	0.821 (0.792–1.000)
Cli	0.741 (0.611–0.857)	0.762 (0.375–0.974)	0.643 (0.414–0.971)
IntraPeriDWI	0.759 (0.630–0.863)	0.714 (0.364–0.951)	0.714 (0.429–1.000)
Cli-IntraPeriDWI	0.844 (0.748–0.928)	0.786 (0.659–0.902)	0.893 (0.786–1.000)

Values are expressed as the area under the receiver operating characteristic curve (AUROC) with 95% confidence interval (CI), as well as sensitivity and specificity with corresponding 95% CIs. Size denotes tumor size, which was modeled as a continuous variable in centimeters. AFP denotes alpha-fetoprotein status, which was modeled as a binary variable using a cutoff of 20 ng/mL: AFP ≤ 20 ng/mL was defined as negative/normal, whereas AFP > 20 ng/mL was defined as elevated/positive. The Cli model was constructed using tumor size and AFP status. The IntraPeriDWI model was constructed using the selected independent imaging predictors, including Tumor_SEM_DDC and P5_DKI_*K*_app_. The Cli-IntraPeriDWI model integrated tumor size, AFP status, Tumor_SEM_DDC, and P5_DKI_*K*_app_

For peritumoral DWI metrics, the top five parameters ranked by diagnostic performance were P3_DKI_*K*_app_ (AUROC = 0.713), P5_DKI_*K*_app_ (AUROC = 0.709), P5_CTRW_α (AUROC = 0.697), P5_IVIM_D_t_ (AUROC = 0.664), and P5_SEM_DDC (AUROC = 0.647) (Fig. 5C).

Considering potential multicollinearity among DWI metrics (reflecting overlapping biophysical characteristics), stepwise logistic regression and VIF filtering (VIF > 5.0) were performed, retaining Tumor_SEM_DDC and P5_DKI_*K*_app_ as independent imaging predictors. As summarized in Table 4, univariate and multivariate logistic regression analyses identified tumor size (odds ratio [OR] = 1.290, 95% confidence interval: 1.003–1.660, *p* = 0.023) and AFP level (OR = 3.142, 95% confidence interval: 1.042–9.475, *p* = 0.040) as independent clinical risk factors for MVI. Subsequently, three models were constructed and compared. The clinical-only model (Cli), incorporating tumor size and AFP, achieved an AUROC of 0.741. The imaging-only model (IntraPeriDWI), incorporating Tumor_SEM_DDC and P5_DKI_*K*_app_, yielded an AUROC of 0.759. The combined model (Cli-IntraPeriDWI), integrating tumor size, AFP, P5_DKI_*K*_app_, and Tumor_SEM_DDC, achieved the highest diagnostic performance (AUROC = 0.844; sensitivity = 0.786; specificity = 0.893) (Fig. 5D). Compared with the clinical-only model, the combined model achieved a significantly higher AUROC for predicting MVI, as demonstrated by the DeLong test (*p* = 0.039), indicating that DWI-derived quantitative features provided incremental predictive value beyond clinical variables alone.

**Table 4:** Univariable and multivariable logistic regression analyses for identifying independent clinical predictors of MVI

Variables	Univariable analysis		Multivariable analysis	
	OR (95% CI)	*p*	OR (95% CI)	*p*
Gender	0.157 (0.019–1.337)	**0.041**	0.193 (0.021–1.819)	0.100
Age	0.966 (0.922–1.013)	0.150		
TBil	0.611 (0.175–2.132)	0.441		
DBil	0.386 (0.098–1.519)	0.168		
ALT	0.938 (0.308–2.850)	0.909		
AST	0.863 (0.263–2.826)	0.808		
GGT	1.875 (0.674–5.213)	0.222		
AFP	4.945 (1.750–13.977)	**0.002**	3.142 (1.042–9.475)	**0.040**
CEA	1.000 (0.156–6.403)	1.000		
CA19-9	1.875 (0.524–6.708)	0.321		
HBV	0.000 (0.000–inf)	0.310		
HCV	0.000 (0.000–inf)	0.053		
Size	1.338 (1.056–1.694)	**0.004**	1.290 (1.003–1.660)	**0.023**
Cirrhosis	1.933 (0.719–5.202)	0.190		

Odds ratios (ORs) are presented with 95% confidence intervals (CIs). Variables with *p* < 0.05 in univariable analysis were entered into the multivariable model. Statistically significant results (*p* < 0.05) are indicated in bold

Furthermore, the calibration curve and confusion matrix (Fig. 5E, F) supported the predictive reliability of the Cli-IntraPeriDWI model in MVI assessment. Detailed diagnostic performance metrics for individual parameters and combined models are presented in Table 3.

### Model interpretability

**Fig. 6:**
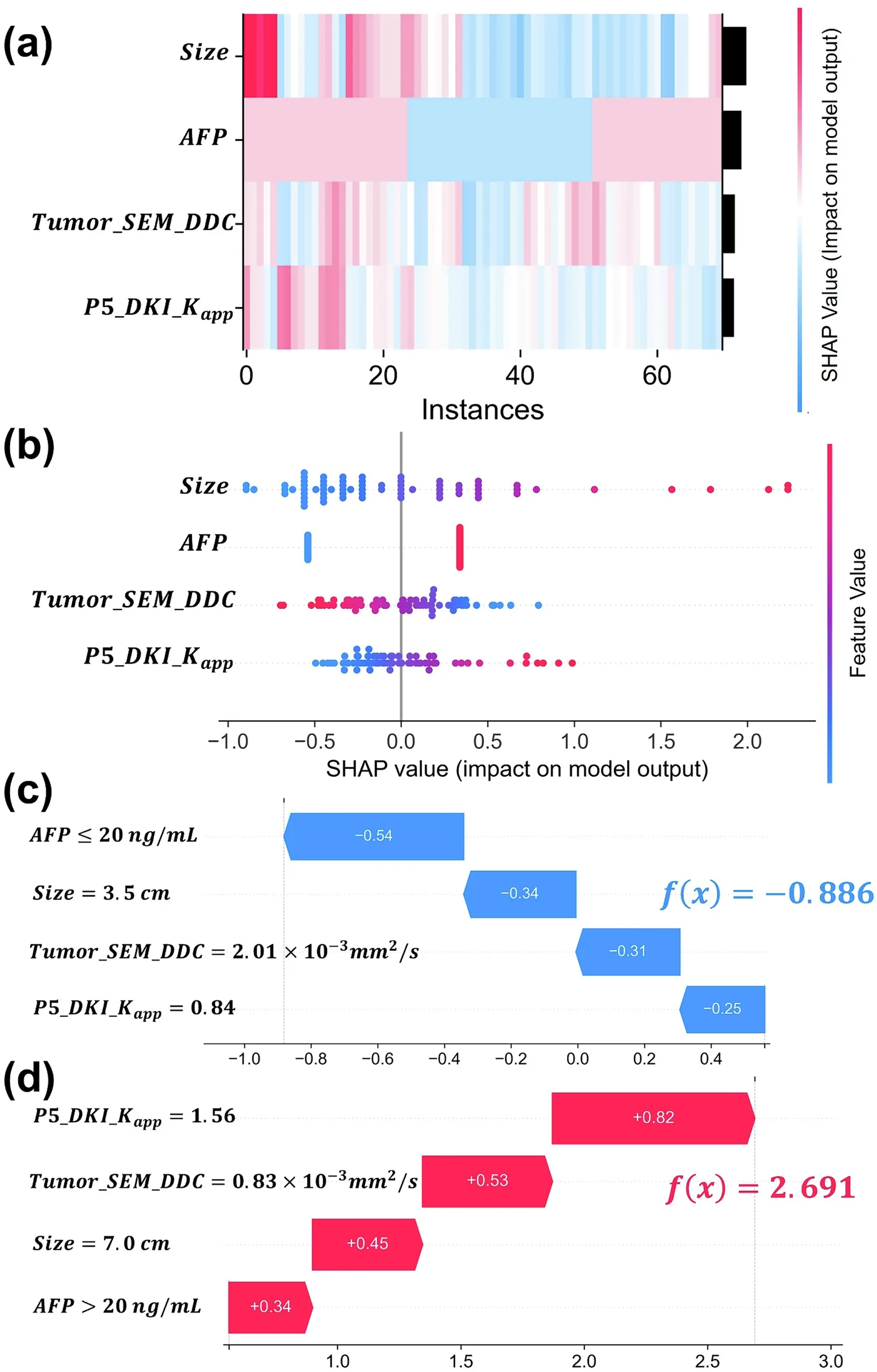
SHAP-based interpretability visualization of the Cli-IntraPeriDWI model. **a** Global SHAP heatmap showing the feature importance across the entire cohort. **b** SHAP summary (beeswarm) plot illustrating the overall contribution and distribution of each feature. **c** SHAP waterfall plot for an individual case predicted as low MVI risk and pathologically confirmed as MVI-negative HCC. **d** SHAP waterfall plot for an individual case predicted as high MVI risk and pathologically confirmed as MVI-positive HCC. AFP, Alpha-fetoprotein; DDC, Distributed diffusion coefficient; DKI, Diffusion kurtosis imaging; *K*ₐₚₚ, Apparent kurtosis coefficient; MVI, Microvascular invasion; SEM, Stretched-exponential model; SHAP, Shapley additive explanations

Figure 6 illustrates the SHAP-based interpretability analysis of the Cli-IntraPeriDWI model. As demonstrated in Fig. 6a, tumor size, AFP, Tumor_SEM_DDC, and P5_DKI_*K*_app_ were the most influential features contributing to the model output. Figure 6b further revealed that larger tumor size, higher AFP levels, lower Tumor_SEM_DDC, and higher P5_DKI_*K*_app_ were positively associated with increased predicted MVI risk. Individual-level SHAP analyses (Fig. 6c, d) highlighted distinct contribution patterns. In patients predicted as low risk, lower AFP (≤ 20 ng/mL), smaller tumor size, relatively higher Tumor_SEM_DDC, and moderate P5_DKI_*K*_app_ collectively exerted negative contributions, thereby reducing the predicted MVI probability. Conversely, in patients predicted as high risk, elevated P5_DKI_*K*_app_ and reduced Tumor_SEM_DDC exhibited strong additive positive effects, which—together with larger tumor size and elevated AFP—substantially increased the predicted likelihood of MVI.

## Discussion

In this prospective study, we systematically integrated multi-scale intratumoral and peritumoral DWI quantitative parameters derived from multiple Gaussian and non-Gaussian diffusion models to investigate their potential as imaging biomarkers for identifying MVI-positive HCC. To our knowledge, this is the first study to comprehensively evaluate multi-scale, multi-model peritumoral DWI quantitative parameters for preoperative MVI prediction in HCC. Our results demonstrated that several peritumoral DWI-derived parameters could serve as potential imaging markers for MVI prediction, with superior diagnostic performance compared with intratumoral metrics. Furthermore, by combining multi-scale intra- and peritumoral DWI parameters with independent clinical risk factors, we developed an interpretable predictive model (Cli-IntraPeriDWI) that remarkably enhanced MVI diagnostic accuracy and provided a transparent decision mechanism, offering a novel, noninvasive approach for precise preoperative MVI prediction.

Regarding intratumoral DWI parameters, MVI-positive HCCs exhibited significantly lower diffusion coefficients, likely reflecting increased nuclear-to-cytoplasmic ratios, higher cellular density, and reduced stromal components in MVI-positive lesions, which collectively restrict water molecular diffusion within the tumor [20]. A similar pattern was observed in peritumoral regions, where diffusion parameters such as P3_IVIM_*D*_t_, P3_DKI_*D*_app_, and P3_SEM_DDC were significantly reduced in MVI-positive cases. Interestingly, several heterogeneity-related DWI parameters also differed significantly between groups, with increased P3_DKI_*K*_app_ and P5_DKI_*K*_app_, accompanied by decreased P3_CTRW_α and P5_CTRW_α. Conceptually, elevated DKI_*K*_app_ and reduced CTRW_α indicate enhanced tissue heterogeneity, reflecting more complex diffusion distributions and microstructural dynamics [21, 22]. Biologically, the increased cellularity and heterogeneity in the peritumoral region likely correspond to intensified tumor infiltration, microvascular remodeling, and reactive fibrosis at the invasive margin of MVI-positive HCC, resulting in a gradual transition from normal hepatic parenchyma to tumor-like tissue [23]. These changes manifest as higher cell density, more intricate microarchitecture, and stronger diffusion restriction in peritumoral DWI [24].

From a diagnostic perspective, Tumor_SEM_DDC exhibited the highest predictive performance among intratumoral DWI parameters. In contrast, four peritumoral parameters—P3_DKI_*K*_app_, P5_DKI_*K*_app_, P5_CTRW_α, and P5_IVIM_*D*_t_—outperformed Tumor_SEM_DDC. Notably, twelve significant peritumoral parameters were identified as potential biomarkers, compared with only four intratumoral parameters. All four intratumoral metrics primarily reflected restricted diffusion, whereas twelve peritumoral metrics captured both restricted diffusion and increased microstructural heterogeneity. These findings suggest that peritumoral diffusion alterations are more sensitive indicators of MVI status. Biologically, this can be explained by the fact that MVI represents a peritumoral pathological phenomenon [15]. In MVI-positive HCC, the tumor–liver interface may harbor microscopic vascular tumor emboli, satellite nodules, microvascular remodeling, increased cellular infiltration, and reactive stromal fibrosis, which together can restrict water diffusion and enhance non-Gaussian diffusion behavior in the peritumoral region [25, 26]. These processes collectively increase local cellularity and disrupt stromal organization, while altering microvascular permeability and extracellular matrix composition, resulting in more pronounced diffusion restriction and non-Gaussian diffusion behavior on DWI [23, 25, 26].

Integrating both intra- and peritumoral DWI quantitative metrics can further enhance MVI diagnostic efficacy. Given the presence of multicollinearity among certain DWI parameters, stepwise logistic regression and VIF filtering were employed to identify independent predictors. Although P3_DKI_*K*_app_ achieved the highest AUROC among individual parameters, P5_DKI_*K*_app_ was retained in the multivariable model, possibly because the 5-mm rim provided a broader and more stable representation of the peritumoral microenvironment with less redundancy relative to Tumor_SEM_DDC and clinical predictors. Ultimately, P5_DKI_*K*_app_ and Tumor_SEM_DDC were retained as independent DWI biomarkers. Consistent with previous studies [27, 28], tumor size and elevated AFP were also identified as independent clinical risk factors for MVI. To address the relative contribution of clinical and DWI-derived predictors, we further compared the Cli-IntraPeriDWI model with a clinical-only model based on tumor size and AFP and an imaging-only model based on Tumor_SEM_DDC and P5_DKI_*K*_app_. This composite model achieved the highest diagnostic accuracy (AUROC = 0.844), outperforming any single DWI metric.

These findings suggest that intratumoral and peritumoral DWI parameters may provide additional diagnostic information beyond conventional clinical factors and may complement tumor size and AFP in preoperative MVI risk assessment. The superior performance of the Cli-IntraPeriDWI model can be attributed to three main aspects: (1) the integration of multi-scale intra- and peritumoral DWI information, capturing complementary features from both tumor core and invasive margin; (2) the inclusion of multiple biophysical attributes reflected by DWI models, encompassing cellularity-related diffusion restriction and heterogeneity-related non-Gaussian diffusion; and (3) the integration of DWI-derived imaging biomarkers with independent clinical risk factors, namely tumor size and AFP, enabling complementary use of imaging phenotypes and clinical characteristics. Furthermore, we performed SHAP-based interpretability analysis to elucidate the decision-making mechanism of the Cli-IntraPeriDWI model. The results revealed that larger tumor size, higher AFP levels, lower Tumor_SEM_DDC, and higher P5_DKI_*K*_app_ were positively associated with increased predicted MVI risk. At the individual level, P5_DKI_*K*_app_ and Tumor_SEM_DDC contributed predominantly to MVI detection sensitivity, while AFP and tumor size played a greater role in maintaining specificity and negative predictive value. Collectively, the complementary interplay between DWI-derived imaging features and clinical biomarkers not only enhanced the model’s overall predictive performance but also provided biologically interpretable insights into MVI prediction.

Several limitations should be acknowledged. First, as a prospective study, patient recruitment was resource-intensive, resulting in a relatively limited sample size. Second, external validation across multiple centers and MRI systems was not performed; thus, the model’s generalizability requires further verification in larger, multicenter, and multi-platform cohorts. Moreover, quantitative DWI parameters may be affected by scanner platform, field strength, gradient performance, *b*-value distribution, signal-to-noise ratio, respiratory motion, distortion correction, and model-fitting algorithms, which may represent important barriers to cross-platform reproducibility and clinical translation. Third, conventional qualitative imaging features—such as arterial enhancement pattern, capsule integrity, and tumor margin configuration—were not incorporated into the current model; future studies integrating both quantitative diffusion metrics and qualitative radiological signs may further improve model performance. Fourth, the peritumoral regions evaluated in this study were limited to two spatial scales, and the influence of different peritumoral extents on biomarker selection and diagnostic performance warrants systematic investigation. Fifth, inter-reader agreement of the DWI-derived quantitative metrics was not assessed because a consensus-based ROI/VOI delineation strategy was used to maximize segmentation accuracy for both intratumoral and automatically generated peritumoral regions. Another limitation is the use of free-breathing DWI with limited signal averaging at higher *b*-values, which may affect parameter stability and reproducibility. Future studies should adopt optimized DWI protocols and include repeatability assessment.

In conclusion, this study systematically evaluated multi-scale peritumoral DWI quantitative parameters derived from multiple diffusion models for predicting MVI in HCC. Compared with intratumoral DWI, peritumoral DWI provided a greater number of potential imaging biomarkers with superior diagnostic capability. By integrating intratumoral and peritumoral DWI parameters with independent clinical risk factors, we developed an interpretable predictive model—Cli-IntraPeriDWI—that achieved optimal predictive performance and transparent decision interpretability for MVI assessment. This model holds promise for noninvasive preoperative MVI risk stratification and individualized treatment planning in HCC, potentially providing useful information for surgical decision-making, postoperative adjuvant treatment consideration, and follow-up intensity. Moreover, our findings suggest that incorporating peritumoral DWI analysis may enhance biomarker sensitivity and diagnostic precision in DWI studies of other solid tumors, potentially yielding substantial clinical benefits.

## Supplementary information

ELECTRONIC SUPPLEMENTARY MATERIAL

## Data Availability

All data generated or analyzed during this study are included in this published article.

## Abbreviations

AFP: Alpha-fetoprotein
AUROC: Area under the receiver operating characteristic curve
Cli-IntraPeriDWI: Combined clinical, intratumoral, and peritumoral diffusion-weighted imaging model
CTRW: Continuous-time random walk model
D_app_ (DKI): Apparent diffusion coefficient (DKI)
DDC (SEM): Distributed diffusion coefficient
DKI: Diffusion kurtosis imaging
*D*_t_ (IVIM): True diffusion coefficient
DWI: Diffusion-weighted imaging
*f* (IVIM): Perfusion fraction
HCC: Hepatocellular carcinoma
IVIM: Intravoxel incoherent motion
*K*_app_ (DKI): Apparent kurtosis coefficient
LI-RADS: Liver imaging reporting and data system
Mono: Monoexponential diffusion model
MVI: Microvascular invasion
ROI: Region of interest
SEM: Stretched-exponential model
SHAP: Shapley additive explanations
VIF: Variance inflation factor
VOI: Volume of interest
α (CTRW): Temporal fractional exponent
α (SEM): Diffusion heterogeneity index
β (CTRW): Spatial fractional exponent
