# Supplementary material for: Preoperative prediction of HCC microvascular invasion by multimodel peritumoral diffusion MRI
Source: Eur Radiol Exp. 2026 Oct 23;10:160. doi: 10.1186/s41747-026-00817-9 (PMC13694582; doi:10.1186/s41747-026-00817-9)
Supplement: Supplementary file 1 [file 41747_2026_817_MOESM1_ESM.pdf]

Preoperative prediction of HCC microvascular invasion by multimodel peritumoral diffusion MRI

ELECTRONIC SUPPLEMENTARY MATERIAL

Supplementary MRI acquisition protocol

All MRI examinations were performed using a 3.0-T commercial scanner (Shanghai United Imaging Healthcare Co., Ltd., Shanghai, China). In addition to the customized multi–b-value DWI sequence described in the main text, routine liver MRI sequences were also acquired for clinical diagnosis and lesion assessment. These included a breath-hold three-dimensional T1-weighted dual-echo gradient-echo (GRE) sequence for in-phase and opposed-phase imaging, with the following parameters: repetition time/echo time (TR/TE), 4.27/1.24 and 2.52 ms; flip angle, 10°; number of axial slices, 70; matrix, 232 × 432; and field of view (FOV), 300 × 434 mm². A T2-weighted fast spin-echo (FSE) sequence was also acquired with the following parameters: TR/TE, 2000/106.2 ms; flip angle, 100°; slice thickness, 6 mm; number of axial slices, 30; matrix, 256 × 256; and FOV, 380 × 380 mm². Dynamic contrast-enhanced imaging was performed using a three-dimensional T1-weighted GRE sequence, with the following parameters: TR/TE, 3.28/1.45 ms; flip angle, 10°; number of axial slices, 60; matrix, 307 × 480; and FOV, 305 × 428 mm².

Supplementary Methods: Diffusion Model Fitting and Parameter Definitions

Voxel-wise diffusion parameters were calculated within each VOI using five diffusion models, including the mono-exponential model, intravoxel incoherent motion (IVIM), diffusion kurtosis imaging (DKI), stretched-exponential model (SEM), and continuous-time random-walk (CTRW) model.

Mono-exponential

For the mono-exponential model, the signal decay was fitted as:

$$\boldsymbol{S(b) = S_0\, e^{-b \cdot ADC}}$$

where $S(b)$ is the signal intensity at a given b-value, S_0 is the signal intensity at b = 0 s/mm^2, and ADC represents the apparent diffusion coefficient. The mono-exponential model mainly reflects the overall water diffusion behavior within tissue and was fitted using b-values of 0, 500, and 800 s/mm^2.

IVIM

For the IVIM model, the signal decay was fitted as:

$$\boldsymbol{S(b) = S_0\left[f\, e^{-b \cdot D_p} + (1-f)\, e^{-b \cdot D_t}\right]}$$

where D_t is the true diffusion coefficient, reflecting pure molecular diffusion; D_p is the pseudo-diffusion coefficient, reflecting microcapillary perfusion-related incoherent motion; and f is the perfusion fraction. The IVIM model allows separation of diffusion-related and perfusion-related components of the DWI signal. Parameter fitting was performed using b-values of 0, 20, 40, 50, 100, 200, 500, and 800 s/mm^2.

DKI

For the DKI model, the signal decay was fitted as:

$$\boldsymbol{S(b) = S_0\, e^{-b \cdot D_{app} + \frac{1}{6} b^2 D_{app}^2 K_{app}}}$$

where D_{app} is the apparent diffusion coefficient derived from the DKI model, and K_{app} is the apparent kurtosis coefficient, which reflects the deviation of water diffusion from Gaussian behavior. A higher K_{app} value suggests a more restricted and heterogeneous diffusion environment. The DKI model was fitted using b-values of 0, 800, 1500, and 2000 s/mm^2.

SEM

For the SEM model, the signal decay was fitted as:

$$S(b) = S_0\, e^{-(b \cdot DDC)^{\alpha}}$$

where DDC is the distributed diffusion coefficient, representing the mean intravoxel diffusion rate, and α in SEM is the diffusion heterogeneity index, with $0 < \alpha \leq 1$. A smaller α indicates greater intravoxel diffusion heterogeneity. The SEM model was fitted using b-values of 0, 100, 200, 500, 800, 1500, and 2000 s/mm^2.

CTRW

For the CTRW model, the signal decay was fitted as:

$$S(b) = S_0\, E_{\alpha}[-(b \cdot D_m)^{\beta}]$$

where $E_{\alpha}(\cdot)$ is the Mittag–Leffler function, D_m is the generalized diffusion coefficient, and α and β in CTRW denote the temporal and spatial fractional exponents, respectively, with $0 < \alpha \leq 1$ and $0 < \beta \leq 1$. Smaller α and β values indicate greater temporal and spatial diffusion heterogeneity, respectively. The CTRW model was fitted using b-values of 0, 100, 200, 500, 800, 1500, and 2000 s/mm^2.

Parameter Definitions and Statistical Analysis For each diffusion model–derived parameter, voxel-wise fitting was first performed within the tumor VOI and the two peritumoral VOIs. The mean value of each parameter within each VOI was then calculated for subsequent statistical analysis. Three spatial regions were analyzed: the intratumoral region, the 3-mm peritumoral rim (P3), and the 5-mm peritumoral rim (P5).

Parameter names were defined using the format **SpatialRegion_Model_Parameter**. For example:

- ADC derived from the mono-exponential model was recorded as **Tumor_Mono_ADC**, **P3_Mono_ADC**, and **P5_Mono_ADC** for the tumor, P3, and P5 regions, respectively.
- Other model-derived parameters were labeled using the same convention, such as **Tumor_SEM_DDC**, **P5_DKI_K$_{app}$**, and **P3_CTRW_α**.
